# Web Usage Data as a Means of Evaluating Public Health Messaging and Outreach

**DOI:** 10.2196/jmir.1278

**Published:** 2009-12-21

**Authors:** Hao Tian, Dana J Brimmer, Jin-Mann S Lin, Abbigail J Tumpey, William C Reeves

**Affiliations:** ^2^Office of the DirectorDivision of Healthcare Quality PromotionCenters for Diseases Control and PreventionAtlantaGAUSA; ^1^Chronic Viral Disease BranchDivision of Viral and Rickettsial DiseasesCenters for Diseases Control and PreventionAtlantaGAUSA

**Keywords:** Internet: Web usage mining, chronic fatigue syndrome, public health campaigns, market basket analysis, Markov chain model, continuing medical education

## Abstract

**Background:**

The Internet is increasingly utilized by researchers, health care providers, and the public to seek medical information. The Internet also provides a powerful tool for public health messaging. Understanding the needs of the intended audience and how they use websites is critical for website developers to provide better services to the intended users.

**Objective:**

The aim of the study was to examine the utilization of the chronic fatigue syndrome (CFS) website at the Centers for Disease Control and Prevention (CDC). We evaluated (1) CFS website utilization, (2) outcomes of a CDC CFS public awareness campaign, and (3) user behavior related to public awareness campaign materials and CFS continuing medical education courses.

**Methods:**

To describe and evaluate Web utilization, we collected Web usage data over an 18-month period and extracted page views, visits, referring domains, and geographic locations. We used page views as the primary measure for the CFS awareness outreach effort. We utilized market basket analysis and Markov chain model techniques to describe user behavior related to utilization of campaign materials and continuing medical education courses.

**Results:**

The CDC CFS website received 3,647,736 views from more than 50 countries over the 18-month period and was the 33rd most popular CDC website. States with formal CFS programs had higher visiting density, such as Washington, DC; Georgia; and New Jersey. Most visits (71%) were from Web search engines, with 16% from non-search-engine sites and 12% from visitors who had bookmarked the site. The public awareness campaign was associated with a sharp increase and subsequent quick drop in Web traffic. Following the campaign, user interest shifted from information targeting consumer basic knowledge to information for health care professionals. The market basket analysis showed that visitors preferred the 60-second radio clip public service announcement over the 30-second one. Markov chain model results revealed that most visitors took the online continuing education courses in sequential order and were less likely to drop out after they reached the Introduction pages of the courses.

**Conclusions:**

The utilization of the CFS website reflects a high level of interest in the illness by visitors to the site. The high utilization shows the website to be an important online resource for people seeking basic information about CFS and for those looking for professional health care and research information. Public health programs should consider analytic methods to further public health by understanding the characteristics of those seeking information and by evaluating the outcomes of public health campaigns. The website was an effective means to provide health information about CFS and serves as an important public health tool for community outreach.

## Introduction

In our fast-paced culture, the Internet has become a common public resource for medical information [1-4]. Survey results from the Pew Internet & American Life Project found that 80% of Internet users looked online for information on health topics [5]. Clinicians also use the Internet to search current information and communicate with patients [6]. In addition to providing information sought by patients and health care providers, the Internet provides a mass medium for health campaigns to generate consumer awareness and influence health behaviors [7].

Optimal use of the Internet for public health messaging requires an understanding of user characteristics, needs, and interests. Websites function as bidirectional communication channels, whereby Web content is the message sent to users and Web usage data from interactions between users and the website represents the visitor feedback. Web usage data reflects users’ contextual interests, geographic locations, and navigation patterns; appropriate analysis provides insight to better understand and serve users’ needs [8,9]. Both the public and private sector have utilized Web usage data to personalize sites, improve website quality, gather business intelligence, and enhance website design based on navigation patterns [9-12].

Chronic fatigue syndrome (CFS) is a debilitating illness of unknown etiology characterized by multiple unexplained symptoms including fatigue [13-15]. CFS affects between 4 and 7 million Americans [16]. A quarter of those with CFS are unemployed or receive disability, and the average family in which a member suffers from the illness foregoes about US$20,000 annually in earnings and wages [17]. In spite of this burden, only half of those with CFS have consulted a physician, and fewer than 20% have been appropriately diagnosed [18-20]. Providing credible evidence-based information concerning CFS to health care providers, patients, and their families has obvious public health significance. In this study, we evaluated the usage of the CDC’s CFS website and focused on three objectives: (1) CFS website utilization, (2) outcomes of a CDC CFS public awareness campaign, and (3) user behavior related to public awareness campaign materials and CFS continuing medical education (CME) courses.

## Methods

The CFS website was launched in June 1996 to provide current evidence-based information about CFS. Since May 2005, CDC websites have used the Omniture Web tracking system (Omniture Inc, Orem, UT, USA). The CDC maintains approximately 300 topic-specific websites. Due to privacy policies, CDC websites do not utilize persistent cookies and cannot collect personal identifiers.

### Data Collection

We based our analysis on website usage data collected over 18 months between June 11, 2006, and December 8, 2007. We selected this time period because the re-designed website was launched on June 11, 2006. The site included four topic segments:

1. “Information for Patients and Caregivers” – basic facts, symptoms, risk factors, diagnosis, treatment options, information for communicating with doctors, and brochures
2. “Information for Healthcare Professionals” – symptoms, diagnosis, treatment options and management plans, toolkits, and brochures
3. “News and Highlights” – new publications, information on the CDC’s CFS public health research program, and an annotated bibliography of peer-reviewed publications
4. “CFS Public Awareness Campaign” – brochures, a photo exhibit, two radio public service announcements (PSAs; 30 and 60 seconds), and a video PSA (30 seconds)

In January 2008, two online CME courses were added to the site: (1) CFS: Diagnosis and Management, for physicians, nurses, and physician assistants, and (2) CFS: A Primer for Allied Health Professionals (see the Multimedia Appendix).

Raw Web usage data were collected and preprocessed by Omniture and then exported in formats of various Web traffic and path reports through SiteCatalyst (Omniture Inc, Orem, UT, USA). We developed a Java program to further process SiteCatalyst reports for specific analyses. Our current analyses excluded visits from CDC computers (ie, CDC staff accessing the site from within the CDC firewall). We also identified and eliminated noisy data in navigation path reports (eg, access by Web crawlers).

### Web Utilization

We used the following information to measure CFS website utilization.

#### Page View

A page view is a view of a full Web page document, which occurred when a visitor opened or reloaded a Web page. Page views to one of the four topic Web pages or an individual page reflected traffic patterns over specific time periods. We defined total page views as the number of times a Web page was viewed in a given period.

#### Visit

A visit is an interaction between a visitor and the website, which occurred when a visitor opened and navigated around the website. In this study, a visit persisted until 30 minutes of inactivity or 12 hours of continuous activity. A single visit could include multiple page views.

#### Geo-Location

Geo-locations are locations in the United States from which visitors accessed the website. We categorized all locations by state. As in other Web usage studies, we excluded AOL (America Online) users from geo-location analysis because their physical geographic positions could not be correctly located by current Web tracking techniques; however, they were included in all other analyses in this study.

#### Visiting Density

The visiting density is the number of page views per Internet population. We estimated the Internet population of each state based on census data [21,22] and calculated state-specific visiting density as follows:

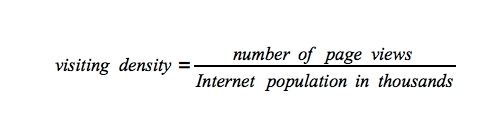



#### Referring Domain

The referring domain is the base domain (without the query string or subdirectories) of the website address that referred a visitor to the CFS website.

We also used views to the publication Web pages to evaluate the utilization of publications on the CFS website.

### Web Traffic Associated With CFS Public Awareness Campaign

On November 6, 2006, the CDC launched a national CFS public awareness campaign with the purpose of educating the public and health care professionals about CFS. The campaign was launched at the National Press Club in Washington, DC and consisted of TV and radio PSAs, press releases, and a traveling public photo exhibit. A major specific aim (and outcome measure) was to encourage utilization of the CFS website. We analyzed the Web usage data around the campaign to describe its impact on Web traffic to the CFS website. We selected three time periods: (1) pre-campaign (5 weeks before campaign: September 17 to October 21, 2006), (2) launch of the campaign (3 weeks around the campaign launch: October 29 to November 18, 2006), and (3) post-campaign (5 weeks after the campaign: November 26 to December 30, 2006). We examined outcomes of the CFS public awareness campaign by analyzing the number of visits to the website and user interests.

### User Behavior Related to CFS Public Awareness Campaign and Online CME Courses

The key to understanding user behavior is to analyze the navigation path, which is the sequence of Web pages a user clicks through while visiting a website. Due to the simple structure of the CFS website, we did not conduct a cluster analysis. Instead, we used a market basket analysis (also known as association rule mining) to inspect the user preference for five different types of media used in the CFS awareness campaign: brochures (in PDF format), a photo exhibit, 30- and 60-second radio PSA clips, and a 30-second video PSA clip. To evaluate user behavior of the two online CME courses, we built a Markov chain model from the navigation paths.

#### Market Basket Analysis

Market basket analysis [23] is a common mathematical technique used by marketing professionals to reveal association rules between individual products or product groups. It has been widely used in retail business to find the relationships between sets of products (ie, purchases) to understand the shopping behavior of customers. The analysis assumes that if you buy certain items in a store, you are more or less likely to buy another type of item. A typical association rule consists of an antecedent and a consequent, which are two disjoint item sets. It is usually measured by the confidence of a rule (scaled from 0% to 100%), which is defined as the ratio of the number of transactions including all items in both antecedent and consequent sets to the number of transactions including only items in the antecedent. An example of such a rule is that 95% (confidence of the association rule) of customers who purchased milk (antecedent) also bought some bread (consequent). The information obtained from such analysis can be used in forming marketing strategies, improving store design for cross-selling, determining promotion and discount plans, and so on.

In this study, we applied market basket analysis to find the visiting associations (ie, association rules), which are the likelihoods of certain pages being viewed together by a visitor. We defined a high association as a confidence rule value of 80% or above. Since we focused only on a small basket of items (ie, less than 10 Web pages), the computation efficiency and excessive irrelevant rules were not issues. We collected navigation paths from the 5 weeks before the campaign launch (September 17 to October 21, 2006) and a 5-week period after the launch (November 26 to December 30, 2006). We transformed navigation paths into “purchasing transactions” consisting of “purchased items” (Web pages with different CFS awareness media types) from a “store” (the CFS website). Each “transaction” made by a “customer” is equivalent to a single navigation path from a visitor. We developed a Java program to implement market basket analysis to describe co-occurrence relationships among usage of different online messaging media types.

#### Markov Chains

A Markov chain is a stochastic process with Markov property [24]. It has been used to model navigation behaviors on websites to predict the next link that a user will click [25], and the chain is defined by a set of states and a set of transitions (ie, the changes of state) between them. Each transition is associated with a probability indicating the likelihood of a transition occurring. In a first-order Markov chain model, the future state depends only on the present state and is independent of past states. In this study, we used a first-order Markov chain model to represent visitors’ navigation information for the two online CME courses and analyze the transition probabilities of the next Web page that a visitor would visit according to what pages they were on. Although this simple Markov chain model ignored the past Web pages viewed by users and calculated the transition probabilities only based on the present Web page, it provided us general navigation patterns. Both of the CME courses consisted of eight components (Syllabus, Introduction, three content-related Chapters, Appendix, Case Study, and References). Each component may contain one Web page (eg, Syllabus and Introduction) or several Web pages (eg, content-related Chapters). We identified and extracted the navigation paths to the courses and used them to build a Markov chain model, whose states and transition probability were defined as follows:

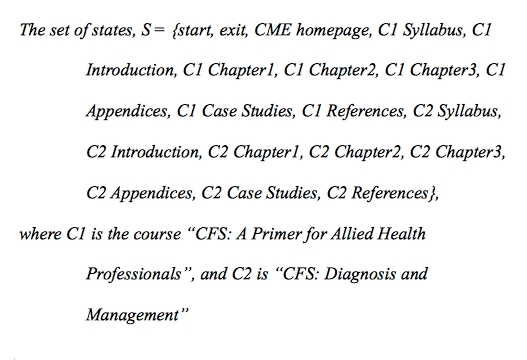

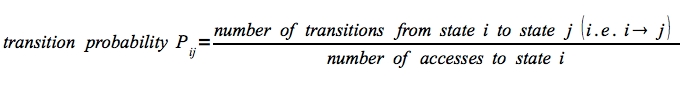





The states in the model included the eight components of each course plus three additional states, *start*, *exit*, and *CME homepage*. The *start* and *exit* states did not correspond to any particular Web page, which only indicated the starting and ending of a visit. By introducing the *exit* state, we defined the dropout probability of a state as the transition probability from this state to *exit* state. *CME homepage* state corresponded to the home Web page of the continuing education portal for the two courses. In other words, we defined “state” as a Web page or group of pages a person views. For example, a visitor could view the Introduction page to a CME course, and this would be considered a state. A transition occurred when a visitor moved from one state to another state (ie, moving from one component within a CME course to another component). The key information in the model is the probability of a transition.

## Results

### Web Utilization

**Figure 1:**
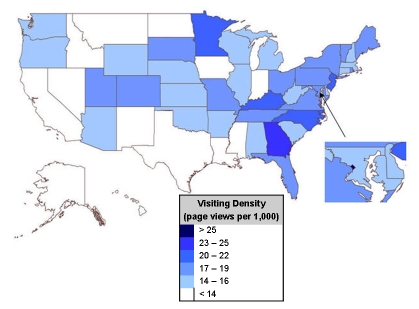
Visiting density of the CFS website

Between June 11, 2006, and December 8, 2007, the CFS website received 843,567 visits, resulting in 3,647,736 page views. During this time period, the CFS website ranked 33rd and was in the top quarter of viewed websites at the CDC. Geographic distribution of page views reflected distribution of the 2007 US population; most visits came from California, Texas, New York, Florida, and Pennsylvania. Of note, 20% of page views came from more than 50 foreign countries. To determine geographic specific interest in CFS, we calculated views per Internet population or state-specific visiting density (Figure 1). Washington, DC had the highest visiting density (111 page views per 1000), followed by Georgia (25 page views per 1000), North Carolina (21 page views per 1000), New Jersey (21 page views per 1000), and Minnesota (20 page views per 1000).

User interest in the four topic segments of the CFS website are shown by aggregating the page views of individual Web pages into topic segments (Table 1). “Information for Patients and Caregivers” had the greatest number of page views (57%), followed by “Information for Healthcare Professionals” (31%), “News and Highlights” (8%), and the “Awareness Campaign” (4%).

**Table 1:** CFS website page views by topic segment

Topic Segment (with top three pages)	No.	%^a^
**Information for patients and caregivers**	**1,432,512**	**57**
CFS basic facts	360,983	14
CFS treatment options	188,852	8
CFS symptoms	127,569	5
**Information for health care professionals**	**774,404**	**31**
CFS diagnostic symptoms	319,544	13
CFS toolkit: fact sheets	114,443	5
CFS treatment	101,008	4
**News and highlights**	**205,504**	**8**
CFS research: new knowledge and publications	54,910	2
CFS publications: new	21,246	1
CFS news and highlights	21,124	1
**Awareness campaign**	**92,412**	**4**
Brochures	36,360	2
Topic segment home page	26,381	1
Public service announcements	9,056	< 1

^a^ Page views to the CFS home page were excluded, resulting in 2,504,832 page views.

Over the 18-month period, the publication section on the CFS website received a total of 199,690 page views. The most frequently viewed page in this section was “New Publications” (76,949 page views), and the top five most frequently viewed papers on the CFS website were accessed 5158, 4709, 4636, 2872, and 2850 times, respectively.

We also assessed how individuals were referred to the website. Among 962,490 visiting instances, 71% (687,316) were referred by Web search engines, 16% (156,142) by non-search-engine websites, and 12% (119,027) directly by bookmarked/typed uniform resource locators (URLs). Among the search engines, Google contributed to 73% of referrals, compared to Yahoo’s 13% and MSN’s 9%.

### CFS Public Awareness Campaign

**Figure 2:**
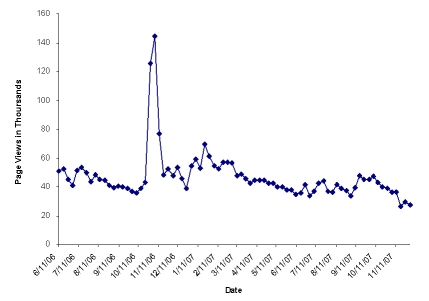
CFS website traffic pattern

After the campaign was launched, the average weekly visits increased more than one and a half times for a 3-week period (from 9594 to 24,977 visits/week) and then dropped to 11,060 visits/week during the 5 weeks post-campaign (Figure 2).

**Figure 3:**
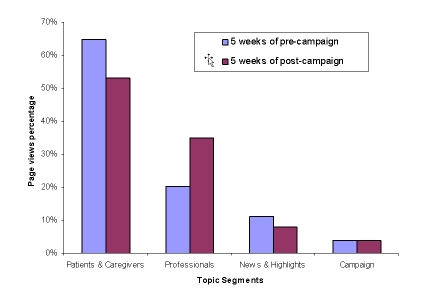
Topic segments and the CFS awareness campaign (y-axis value is the percentage of page views to a topic segment over the total page views to the website; page views to the home page are excluded)

In addition to the change in Web traffic volume, the proportion of page views to the four topic segments also changed following launch of the campaign (Figure 3). Although the percentage of page views to the topic segment “Information for Patients and Caregivers” remained the highest, it decreased from 65% to 53%, whereas the percentage of page views to “Information for Healthcare Professionals” increased from 20% before the campaign to 35% after the campaign.

### User Behavior for CFS Campaign Materials and CME Courses

**Figure 4:**
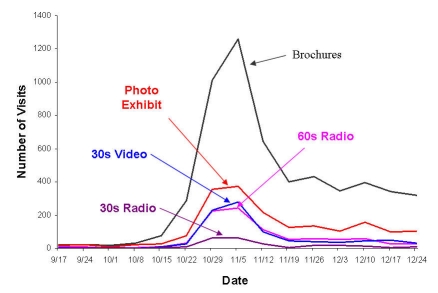
Usage of the different CFS campaign materials

Figure 4 depicts visitors’ preferences for the different types of CFS campaign materials. The spike indicates the time period of the campaign launch. After the campaign launch, the brochure was the most frequently viewed campaign media type followed by the photo exhibit. The 60-second radio clip and 30-second video clip had approximately an equal number of visits, whereas the 30-second radio clip was the least viewed type of media.

**Figure 5:**
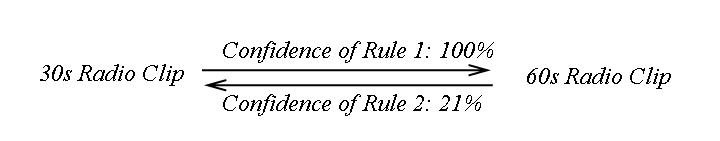
Association Rules 1 and 2

**Figure 6:**
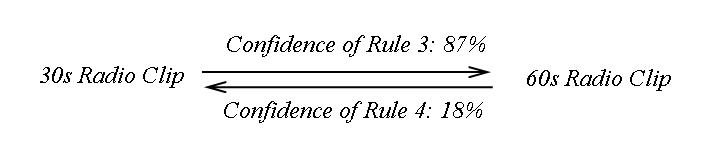
Association Rules 3 and 4

Market basket analysis results indicated users’ preference for the 60-second radio clip over the 30-second radio clip (Figure 5 and Figure 6) as found in the 5 weeks prior to launch and post-campaign. The association rules between other types of campaign materials such as brochures vs photo exhibit, brochures vs video, video vs 60-second radio clip, etc, all had low confidence values ranging from 1 to 65, well below the cutoff of 80.

Association Rule 1 shows that in the 5 weeks before the campaign launch, 100% of visitors who viewed the 30-second PSA radio clip page also viewed the 60-second PSA radio clip page, compared to 21% of visitors who viewed the 60-second PSA radio clip page and then also viewed the 30-second PSA radio clip page (Association Rule 2).

In the 5 weeks after the campaign launch, 87% of visitors who viewed the 30-second PSA radio clip page also viewed the 60-second PSA radio clip page (Association Rule 3), vs 18% of visitors who viewed the 60-second PSA radio clip page and then viewed the 30-second PSA radio clip page (Association Rule 4).Thus, overwhelmingly, visitors who viewed the 30-second PSA radio clip page also viewed the 60-second PSA radio clip page, yet only a small proportion of visitors who viewed the 60-second radio clip (21%) checked the 30-second radio clip as well (18%).

**Figure 7:**
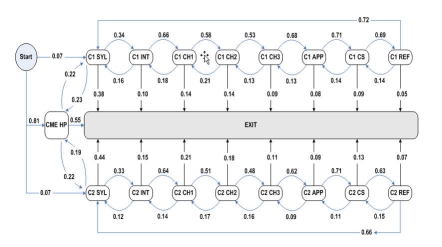
Markov chain model of online CFS CME courses (ovals represent the states and arrows represent the transitions; the number next to each arrow is the transition probability; transition probabilities less than 0.05 are not shown; C1: CFS – A Primer for Allied Health Professional; C2: CFS – Diagnosis and Management)

From January 1 to August 31, 2008, there were 43,428 page views to the online CFS CME courses (Multimedia Appendix: i. Continuing Education Portal). From these page views, 8070 navigation paths were identified and used to build the Markov chain model (Figure 7).

For all visits (n = 8070), 46% (3737) viewed the home page and then exited. The Diagnosis and Management CME course received 2451 (30%) visits and the A Primer for Allied Health Professional course, 2298 (28%). Only 5% (407) viewed the content from both courses. The user visiting patterns identified by the Markov chain model matched the Web structure of the two courses very well. All transitions between two course components could match to either a “next page” or “previous page” button on the Web pages, indicating that users followed the courses in sequential order. Although users could directly access any component from anywhere in the course through the left navigation panel on each page, the probabilities of these skip patterns or short-cut transitions were shown to be not greater than 0.05 in the Markov chain model. The Markov chain model also found that the dropout probabilities from the CME home page and Syllabus pages were much higher than those from other course components. The CME home page was the most common entrance (0.81) and exit (0.55) to the CME courses. The transition probabilities from the CME home page to the Syllabus pages of the two courses were the same (0.22). It is to some extent unexpected that the dropout probabilities on the Syllabus pages were high (0.38/0.44). Based on the structure of the Syllabus Web pages, it is possible that this high dropout rate reflects the fact that the hyperlink to the Introduction page is at the bottom of the page and is not highlighted, in addition to the excessive length of these pages (> 120 lines).

## Discussion

The high utilization rate of the CDC CFS website indicates the magnitude of interest in CFS and reflects the website’s importance as an online resource for investigators, health care providers, patients, and caregivers around the world. Over 6000 page views to the website occurred each day, with over 840,000 visits over an 18-month period.

Analysis of geographic-specific CFS website utilization provided important information. Simply tabulating geographic distribution of website use by state is misleading since this merely reflects the US population. Visiting density is a better index that indicates the likelihood of individuals visiting the site based on the Internet population. Washington, DC and Georgia had the highest visiting densities and are home to the US Department of Health and Human Services and the CDC. North Carolina is the headquarters for the Chronic Fatigue and Immune Dysfunction Syndrome Association of America, a large patient advocacy group and CDC’s contractor managing the public awareness campaign. New Jersey is home to the University of Medicine & Dentistry of New Jersey, sponsoring one of the largest and highly respected CFS research programs, and to the New Jersey Chronic Fatigue Syndrome Association, an active patient advocacy group. Finally, the Mayo Clinic Hospital, which has a CFS clinical program, is located in Minnesota, the state with the fifth highest visiting density. Visiting density indicates the potential importance of CFS research institutions in directing visitors to the CDC CFS website.

Analysis of referral sources to the website also revealed important information. Not surprisingly, the Google search engine sent most visitors to the CFS website, followed by Yahoo and MSN. Twelve percent of visitors bookmarked the CFS Web address. It would be of interest to determine what proportion stemmed from patient advocacy groups or persons conducting CFS research.

The website contains a section with all publications from the CDC CFS public health research program and receives a considerable amount of views compared to online journals. For example, our five most popular publications were viewed between 5158 and 2850 times. This is similar to the top 10 most-viewed articles published in 2008 by BMC Genomics, which were accessed between 8066 and 2880 times.

Evaluation of website use also provides quantitative data concerning the effectiveness of messaging in public health campaigns and their sustainability. The sharp spike in Web visits in early November 2006 revealed a boost in campaign exposure related to a half day Department of Health and Human Services event at the National Press Club to kick off the CFS public awareness campaign. However, the spike decreased after 3 weeks and ultimately reverted to levels before the campaign. The timeline illustrates that a campaign booster inserted 2 to 3 months after the launch may be appropriate to sustain interest. Many campaigns initiate a boost 6 to 12 months after the initial launch, and, based on our results, this timeframe may delay potential sustainability benefits in terms of website utilization.

Web usage data provide valuable information concerning how websites can attract more visitors. The most-viewed Web pages represent the users’ interests and should be easy to access and frequently updated, and this should take into account shifts in interest. Comparing the 5 weeks before and 5 weeks after launch of the campaign, the “Information for Healthcare Professionals” segment showed the greatest percentage increase in page views (15% increase), and the “Information for Patients and Caregivers” section had the greatest percentage decrease in views (12% decrease). While we cannot identify the audience looking at these sections, one possible explanation is that return visitors started looking at CFS professional information rather than just the information for patients. Alternatively, there could have been an increase in new visitors looking for professional health care information. Regardless of which segment a visitor accessed, we found that the CDC CFS website was most frequently visited by individuals seeking basic facts, treatment options, symptoms, and publications.

When we examined user behavior regarding the campaign materials, the brochure was the most popular media type among visitors and consistently sustained a higher level of page visits than all other campaign materials. All brochures on the website are designed to be printed, and this may help to explain why people preferred the brochure—it is a tool that visitors can take with them. The longer radio clip (60 seconds) performed much better than the shorter one (30-second radio or video clip). The market basket analysis showed that none of the visitors listened to the 30-second radio clip without checking out the 60-second radio clip during the 5 weeks after the clips were put on the Internet, while the majority of visitors to the 60-second radio clip did not access the 30-second radio clip. This suggests the public’s preference for a longer media clip in terms of CFS information, perhaps because the shorter radio clip did not provide enough information. Public health campaigns using the Internet may want to consider utilizing market basket analysis techniques to improve campaign evaluations and sustainability efforts as these analyses help to determine which components are actually viewed by the public or target audience. For example, the cost-effectiveness implication from this study is that campaign planners may want to consider not running both 30-second and 60-second radio PSAs during the same campaign time period. Instead, organizers may want to start with the 60-second spot and present the 30-second advertisement in a campaign booster. In addition, one could run market basket analyses on a more frequent basis (ie, every 2 months) to monitor the usage-association changes among campaign materials or Web pages in order to improve the campaign sustainability.

The match between the user visiting pattern in the Markov chain model and the Web structure of the two CME courses reflects the fact that the users took the courses in the sequential order recommended by the website. The two courses are designed for different audiences, and Markov chain model results show that only 5% of visitors visited each course in the same visit, indicating that each of the courses is serving a different population. The Web traffic volume and navigation patterns to them are very similar for both courses. The high dropout probabilities for the CME home page and two Syllabus pages imply that visitors were less likely to leave the course after they reached the Introduction pages. In other words, persons who do start the course are likely to finish it.

As shown by the Markov chain model results, factors were identified that may affect sustainability of visits to a website. Public health websites may want to apply the Markov chain analysis to all content on a website to identify the main exit points and therefore improve Web structure and content. When visitors stay longer on a site, it increases the probability of exposure to information.

### Limitations

This study has several limitations. The CDC website allows only session cookies, not persistent cookies; therefore, we cannot get the accurate number of new/unique visitors to provide more insightful information than the number of visits/page views. Theoretically, one individual could access the website frequently, but results of both the geographic pages views and visiting density analysis reduce the likelihood of this possibility. AOL users were excluded in the geographic distribution analysis given the technical issues of identifying their geographic locations. Despite this, the number of page views from AOL users accounted for only 4% of total page views to the website, and exclusion of this data should likely not have a significant impact.

The user behavior analysis in this study focuses on only a particular group of Web pages such as campaign media pages and CME courses, and user navigation analysis of the campaign materials was limited to 5 weeks pre- and post-campaign launch, which did not allow for tracking user behavior over the 18-month period. However, the decision to analyze time periods was determined a priori and centered on the campaign launch dates, which allows for a more accurate and narrow timeframe of comparison. The CDC CFS website has a simple hierarchical Web structure, with most content at levels 3 and 4. Conducting a broad range of behavior analyses on data collected from such a simple website may cover or hide the issues that we found in this study through a more topic-focused behavior analysis. However, defining an appropriate level for the comprehensive market basket and Markov chain analyses of the whole website will be of interest to all public health website managers.

### Future Research

All CDC websites are periodically updated to reflect current developments in the Internet as well as content information. Currently, the CDC CFS website is undergoing an upgrade with a new template, and findings from this study will provide valuable information to the reconstruction of the new site. Once the new website has been completed, market basket and Markov chain analyses will be conducted to compare the results of the two different Web designs. We hypothesize that a better understanding of the impacts of different public health Web structures can be obtained. We also plan to apply navigation pattern analysis to the entire website. The interaction analysis can also be enhanced by increasing the order of the Markov chain model. As a result, the transition probability will be determined by a certain number of past states, rather than just the present state.

### Conclusion

This study shows that the CFS website is an important online resource for the public regarding CFS, especially the topics of basic facts, symptoms, and treatments. The popularity of CFS publications on the website to some extent reflects the significant position of public health agencies in the field of CFS research. The market basket analysis, a traditional analytic technique in retail business, was applied to this public health website and showed utility in identifying user preferences for different online public health messaging formats. Markov chain analysis confirmed that visitors completed the CME courses in sequential order. In summary, the CFS website is an effective way of providing CFS health education and information and serves an important tool in public health outreach.

**Acknowledgments**

This study was fully funded by the US Centers for Disease Control and Prevention, Atlanta, GA, USA. The findings and conclusions in this report are those of the authors and do not necessarily represent the views of the funding agency.

The authors would like to thank Wies Rafi and Walter F Smith, National Center for Health Marketing, and Anthony McDonald, National Center for Public Health Informatics, for helping in data collection and processing.

## Multimedia Appendix

Screenshots of some CDC CFS Web pages

## Abbreviations

AOL: America Online
CDC: Centers for Disease Control and Prevention
CFS: chronic fatigue syndrome
CME: continuing medical education
PSA: public service announcement
