# Supplementary material for: Web Usage Data as a Means of Evaluating Public Health Messaging and Outreach
Source: J Med Internet Res. 2009 Dec 21;11(4):e52. doi: 10.2196/jmir.1278 (PMC2802568; doi:10.2196/jmir.1278)
Supplement: Supplementary file 1 [file jmir_v11i4e52_app1.pdf]

Multimedia Appendix 1.
Screenshots of some CDC CFS web pages

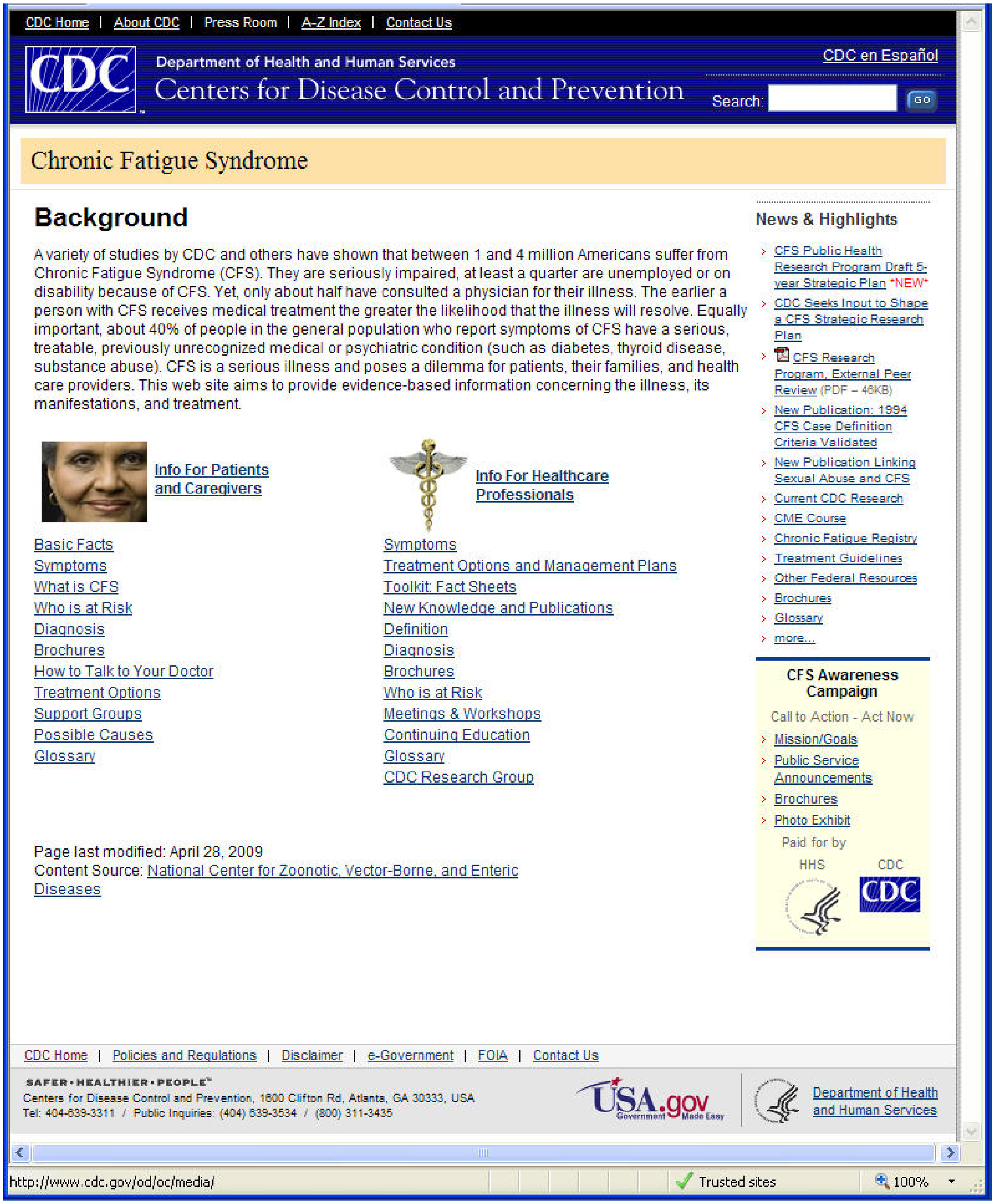

a. Screenshot of the web site homepage

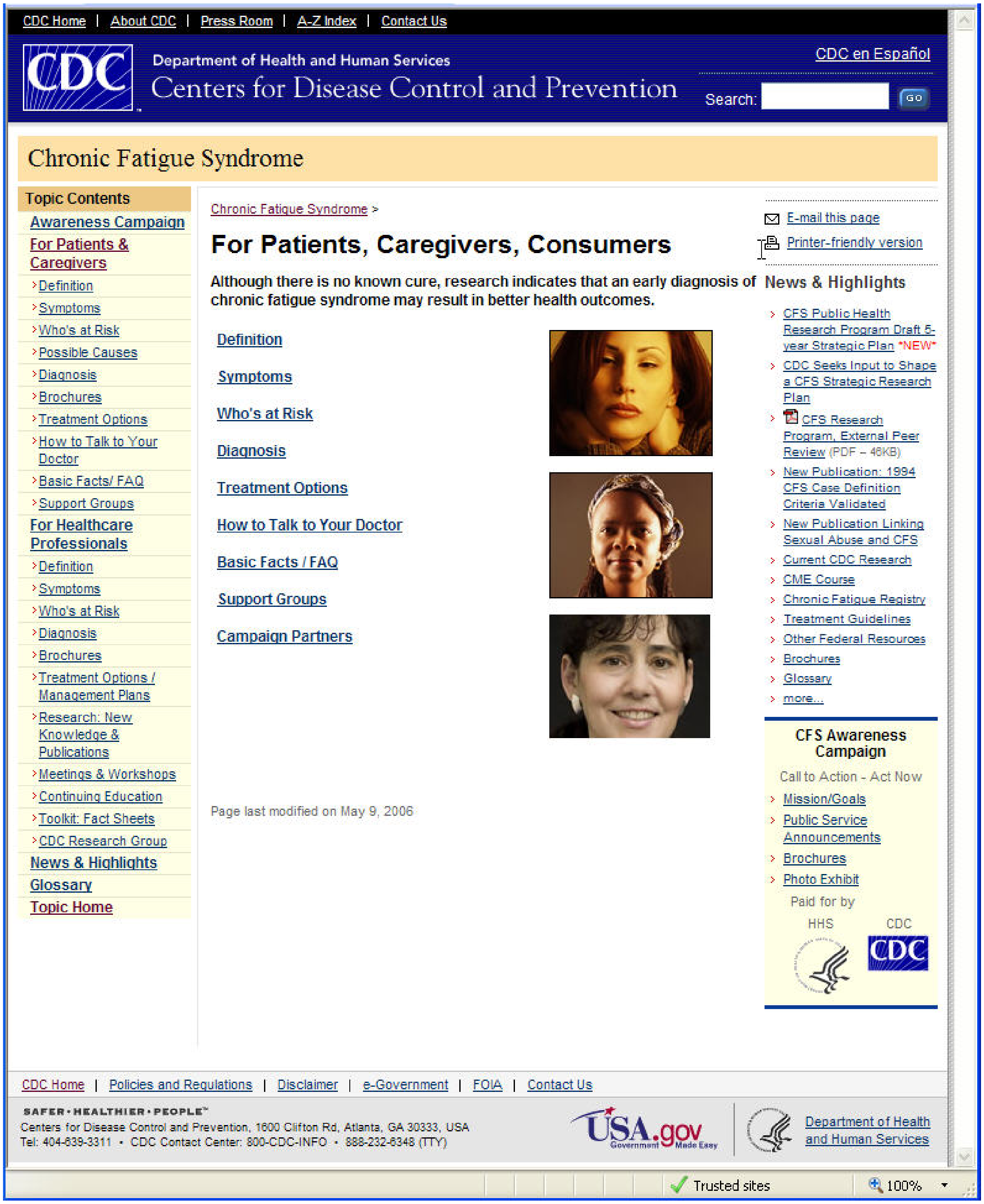
CDC Home | About CDC | Press Room | A-Z Index | Contact Us

CDC — Department of Health and Human Services — Centers for Disease Control and Prevention

CDC en Español

Search: GO

Chronic Fatigue Syndrome

Topic Contents
- Awareness Campaign
- For Patients & Caregivers
  - Definition
  - Symptoms
  - Who's at Risk
  - Possible Causes
  - Diagnosis
  - Brochures
  - Treatment Options
  - How to Talk to Your Doctor
  - Basic Facts/ FAQ
  - Support Groups
- For Healthcare Professionals
  - Definition
  - Symptoms
  - Who's at Risk
  - Diagnosis
  - Brochures
  - Treatment Options / Management Plans
  - Research: New Knowledge & Publications
  - Meetings & Workshops
  - Continuing Education
  - Toolkit: Fact Sheets
  - CDC Research Group
- News & Highlights
- Glossary
- Topic Home

Chronic Fatigue Syndrome >

For Patients, Caregivers, Consumers

Although there is no known cure, research indicates that an early diagnosis of chronic fatigue syndrome may result in better health outcomes.

- Definition
- Symptoms
- Who's at Risk
- Diagnosis
- Treatment Options
- How to Talk to Your Doctor
- Basic Facts / FAQ
- Support Groups
- Campaign Partners

Page last modified on May 9, 2006

E-mail this page
Printer-friendly version

News & Highlights
- CFS Public Health Research Program Draft 5-year Strategic Plan *NEW*
- CDC Seeks Input to Shape a CFS Strategic Research Plan
- CFS Research Program, External Peer Review (PDF – 46KB)
- New Publication: 1994 CFS Case Definition Criteria Validated
- New Publication Linking Sexual Abuse and CFS
- Current CDC Research
- CME Course
- Chronic Fatigue Registry
- Treatment Guidelines
- Other Federal Resources
- Brochures
- Glossary
- more...

CFS Awareness Campaign
Call to Action - Act Now
- Mission/Goals
- Public Service Announcements
- Brochures
- Photo Exhibit

Paid for by
HHS CDC

CDC Home | Policies and Regulations | Disclaimer | e-Government | FOIA | Contact Us

SAFER · HEALTHIER · PEOPLE™
Centers for Disease Control and Prevention, 1600 Clifton Rd, Atlanta, GA 30333, USA
Tel: 404-639-3311 • CDC Contact Center: 800-CDC-INFO • 888-232-6348 (TTY)

USA.gov Government Made Easy

Department of Health and Human Services

Trusted sites 100%

b. Screenshot of navigation web page for the topic segment:
Information for Patients and Caregivers

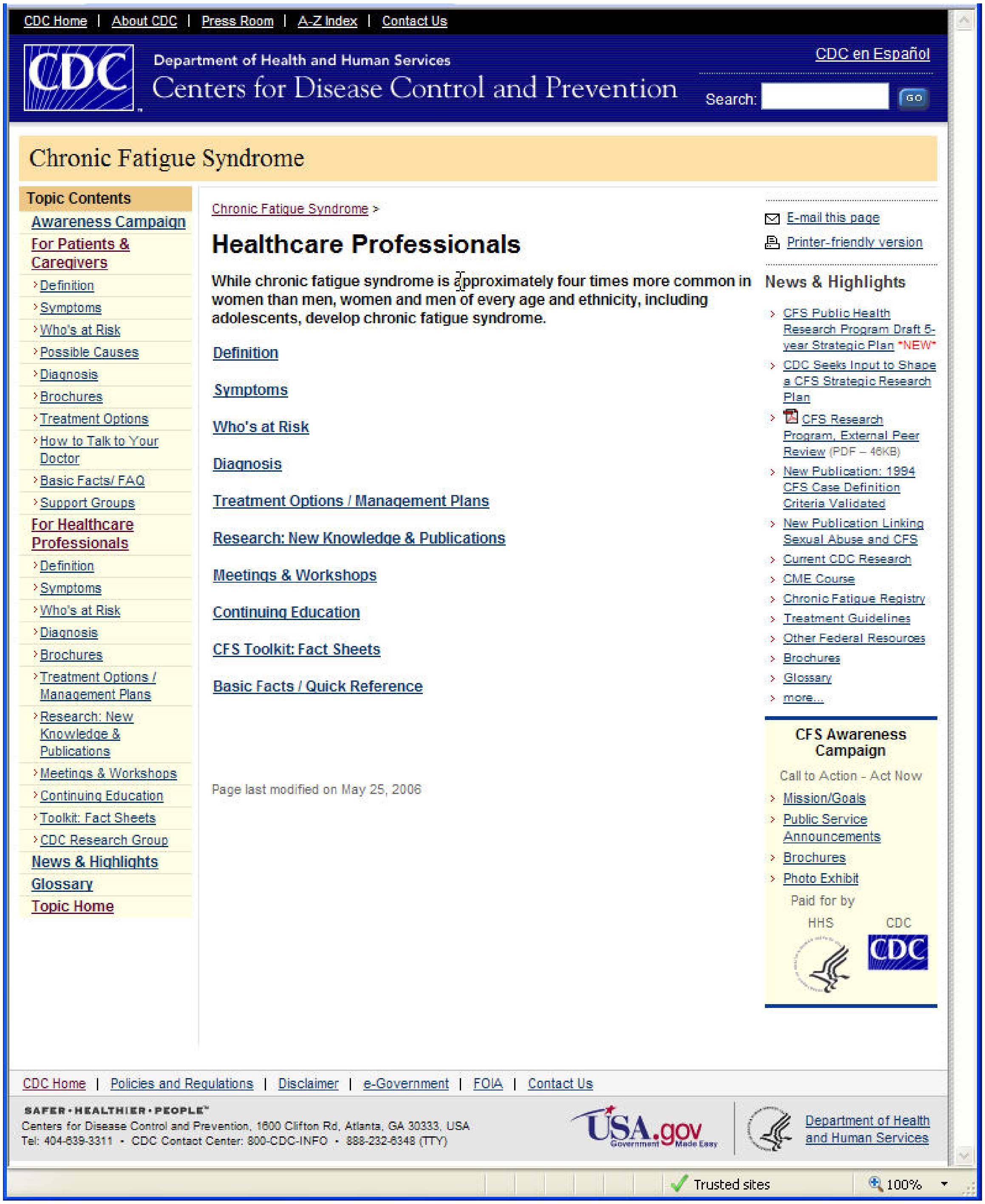

c. Screenshot of navigation web page for the topic segment:
Information for Healthcare Professionals

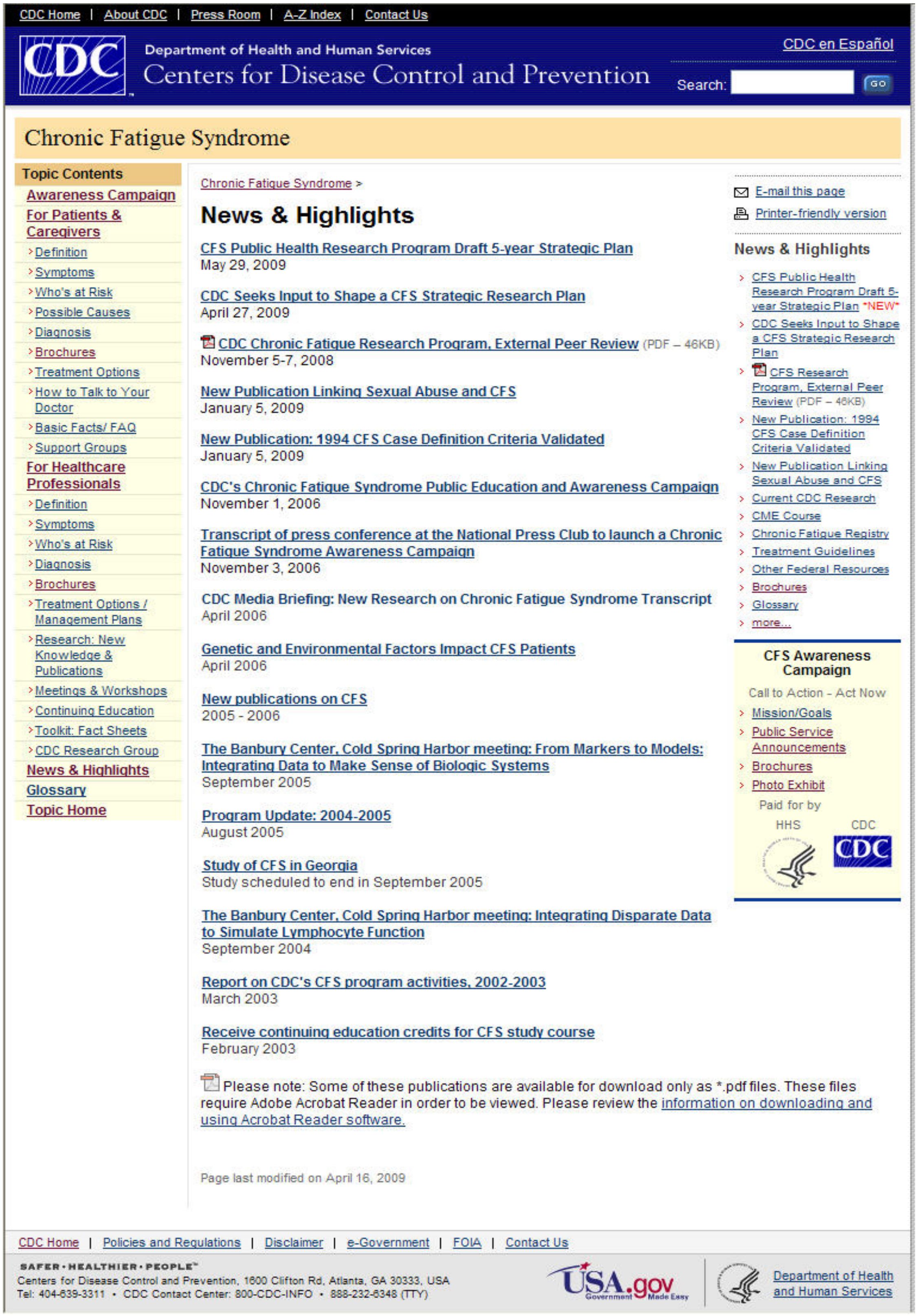

CDC Home | About CDC | Press Room | A-Z Index | Contact Us

Department of Health and Human Services
Centers for Disease Control and Prevention

CDC en Español

Search: GO

Chronic Fatigue Syndrome

Topic Contents

- Awareness Campaign
- For Patients & Caregivers
  - Definition
  - Symptoms
  - Who's at Risk
  - Possible Causes
  - Diagnosis
  - Brochures
  - Treatment Options
  - How to Talk to Your Doctor
  - Basic Facts/ FAQ
  - Support Groups
- For Healthcare Professionals
  - Definition
  - Symptoms
  - Who's at Risk
  - Diagnosis
  - Brochures
  - Treatment Options / Management Plans
  - Research: New Knowledge & Publications
  - Meetings & Workshops
  - Continuing Education
  - Toolkit: Fact Sheets
  - CDC Research Group
- News & Highlights
- Glossary
- Topic Home

Chronic Fatigue Syndrome >

News & Highlights

CFS Public Health Research Program Draft 5-year Strategic Plan
May 29, 2009

CDC Seeks Input to Shape a CFS Strategic Research Plan
April 27, 2009

CDC Chronic Fatigue Research Program, External Peer Review (PDF – 46KB)
November 5-7, 2008

New Publication Linking Sexual Abuse and CFS
January 5, 2009

New Publication: 1994 CFS Case Definition Criteria Validated
January 5, 2009

CDC's Chronic Fatigue Syndrome Public Education and Awareness Campaign
November 1, 2006

Transcript of press conference at the National Press Club to launch a Chronic Fatigue Syndrome Awareness Campaign
November 3, 2006

CDC Media Briefing: New Research on Chronic Fatigue Syndrome Transcript
April 2006

Genetic and Environmental Factors Impact CFS Patients
April 2006

New publications on CFS
2005 - 2006

The Banbury Center, Cold Spring Harbor meeting: From Markers to Models: Integrating Data to Make Sense of Biologic Systems
September 2005

Program Update: 2004-2005
August 2005

Study of CFS in Georgia
Study scheduled to end in September 2005

The Banbury Center, Cold Spring Harbor meeting: Integrating Disparate Data to Simulate Lymphocyte Function
September 2004

Report on CDC's CFS program activities, 2002-2003
March 2003

Receive continuing education credits for CFS study course
February 2003

Please note: Some of these publications are available for download only as *.pdf files. These files require Adobe Acrobat Reader in order to be viewed. Please review the information on downloading and using Acrobat Reader software.

Page last modified on April 16, 2009

E-mail this page
Printer-friendly version

News & Highlights

- CFS Public Health Research Program Draft 5-year Strategic Plan *NEW*
- CDC Seeks Input to Shape a CFS Strategic Research Plan
- CFS Research Program, External Peer Review (PDF – 46KB)
- New Publication: 1994 CFS Case Definition Criteria Validated
- New Publication Linking Sexual Abuse and CFS
- Current CDC Research
- CME Course
- Chronic Fatigue Registry
- Treatment Guidelines
- Other Federal Resources
- Brochures
- Glossary
- more...

CFS Awareness Campaign

Call to Action - Act Now

- Mission/Goals
- Public Service Announcements
- Brochures
- Photo Exhibit

Paid for by
HHS CDC

CDC Home | Policies and Regulations | Disclaimer | e-Government | FOIA | Contact Us

SAFER·HEALTHIER·PEOPLE™
Centers for Disease Control and Prevention, 1600 Clifton Rd, Atlanta, GA 30333, USA
Tel: 404-639-3311 • CDC Contact Center: 800-CDC-INFO • 888-232-6348 (TTY)

USA.gov Government Made Easy

Department of Health and Human Services

d. Screenshot of navigation web page for the topic segment:
News and Highlights

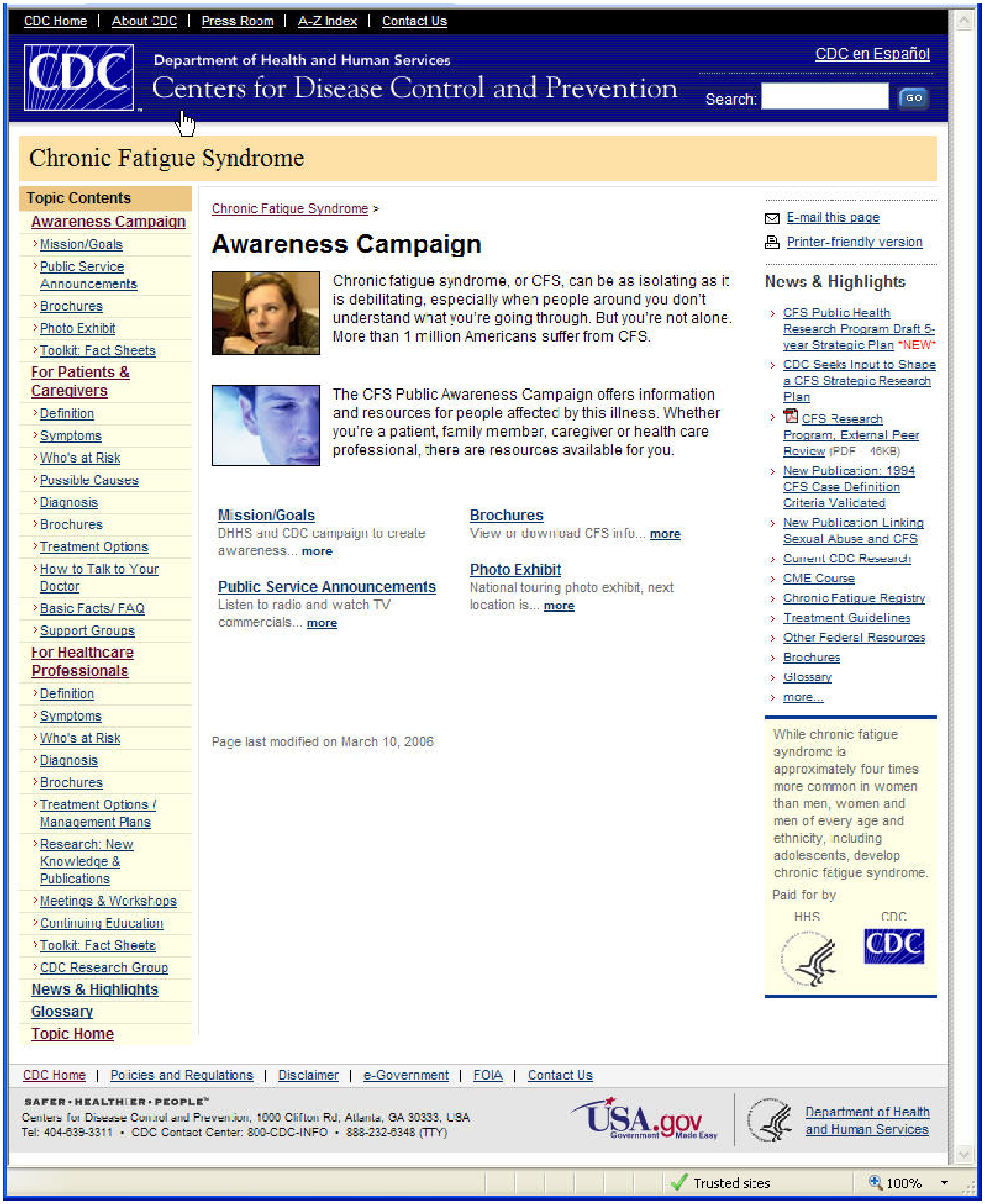

e. Screenshot of navigation web page for the topic segment:
CFS Public Awareness Campaign

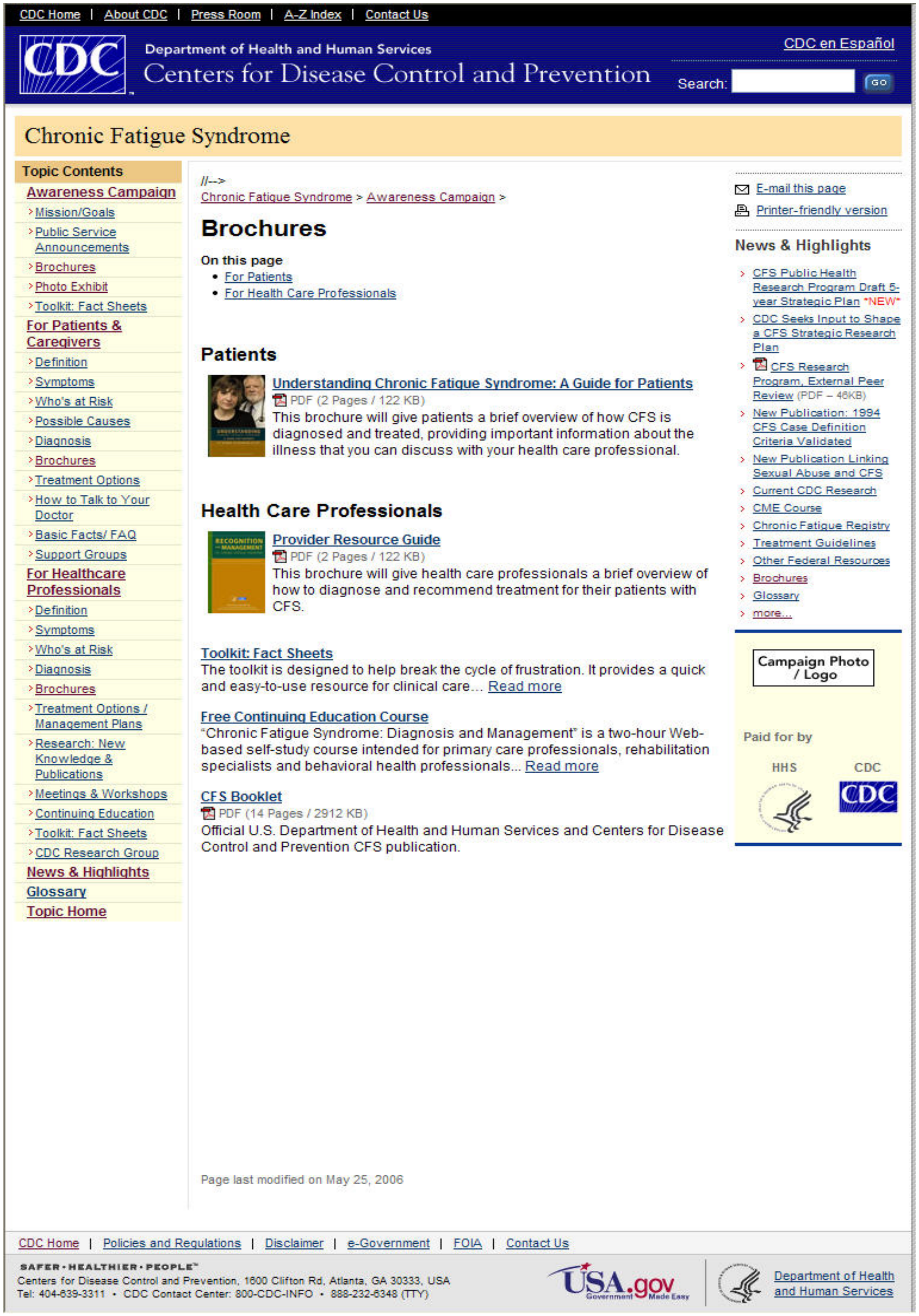

CDC Home | About CDC | Press Room | A-Z Index | Contact Us

Department of Health and Human Services
Centers for Disease Control and Prevention

CDC en Español

Search: GO

Chronic Fatigue Syndrome

Topic Contents

- Awareness Campaign
  - Mission/Goals
  - Public Service Announcements
  - Brochures
  - Photo Exhibit
  - Toolkit: Fact Sheets
- For Patients & Caregivers
  - Definition
  - Symptoms
  - Who's at Risk
  - Possible Causes
  - Diagnosis
  - Brochures
  - Treatment Options
  - How to Talk to Your Doctor
  - Basic Facts/ FAQ
  - Support Groups
- For Healthcare Professionals
  - Definition
  - Symptoms
  - Who's at Risk
  - Diagnosis
  - Brochures
  - Treatment Options / Management Plans
  - Research: New Knowledge & Publications
  - Meetings & Workshops
  - Continuing Education
  - Toolkit: Fact Sheets
  - CDC Research Group
- News & Highlights
- Glossary
- Topic Home

//-->
Chronic Fatigue Syndrome > Awareness Campaign >

Brochures

On this page
- For Patients
- For Health Care Professionals

Patients

Understanding Chronic Fatigue Syndrome: A Guide for Patients
PDF (2 Pages / 122 KB)
This brochure will give patients a brief overview of how CFS is diagnosed and treated, providing important information about the illness that you can discuss with your health care professional.

Health Care Professionals

Provider Resource Guide
PDF (2 Pages / 122 KB)
This brochure will give health care professionals a brief overview of how to diagnose and recommend treatment for their patients with CFS.

Toolkit: Fact Sheets
The toolkit is designed to help break the cycle of frustration. It provides a quick and easy-to-use resource for clinical care… Read more

Free Continuing Education Course
"Chronic Fatigue Syndrome: Diagnosis and Management" is a two-hour Web-based self-study course intended for primary care professionals, rehabilitation specialists and behavioral health professionals... Read more

CFS Booklet
PDF (14 Pages / 2912 KB)
Official U.S. Department of Health and Human Services and Centers for Disease Control and Prevention CFS publication.

E-mail this page
Printer-friendly version

News & Highlights
- CFS Public Health Research Program Draft 5-year Strategic Plan *NEW*
- CDC Seeks Input to Shape a CFS Strategic Research Plan
- CFS Research Program, External Peer Review (PDF – 46KB)
- New Publication: 1994 CFS Case Definition Criteria Validated
- New Publication Linking Sexual Abuse and CFS
- Current CDC Research
- CME Course
- Chronic Fatigue Registry
- Treatment Guidelines
- Other Federal Resources
- Brochures
- Glossary
- more...

Campaign Photo / Logo

Paid for by
HHS CDC

Page last modified on May 25, 2006

CDC Home | Policies and Regulations | Disclaimer | e-Government | FOIA | Contact Us

SAFER · HEALTHIER · PEOPLE™
Centers for Disease Control and Prevention, 1600 Clifton Rd, Atlanta, GA 30333, USA
Tel: 404-639-3311 • CDC Contact Center: 800-CDC-INFO • 888-232-6348 (TTY)

USA.gov Government Made Easy
Department of Health and Human Services

f. Screenshot of web page: *Brochures*

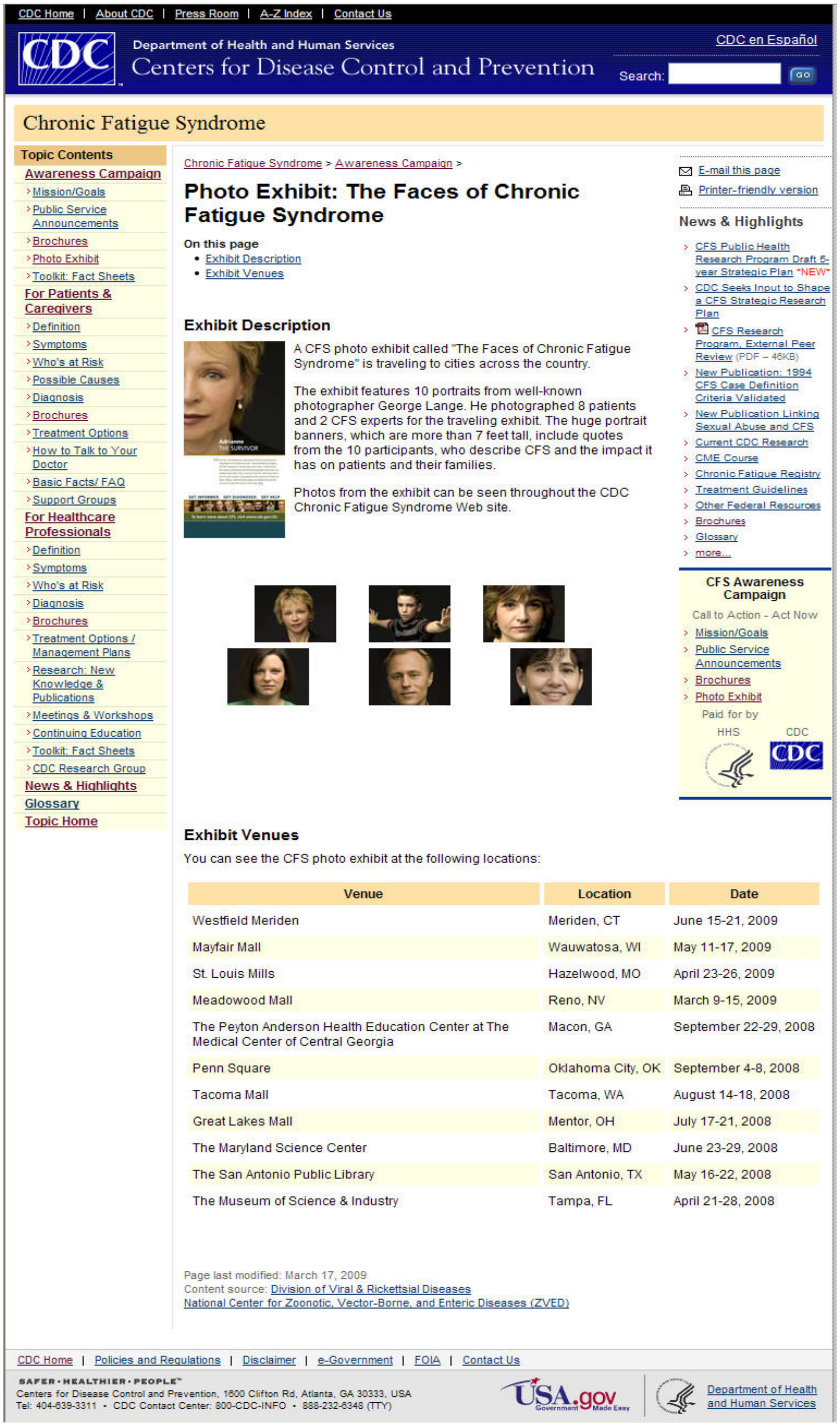

CDC Home | About CDC | Press Room | A-Z Index | Contact Us

Department of Health and Human Services
Centers for Disease Control and Prevention

CDC en Español

Search: GO

Chronic Fatigue Syndrome

Topic Contents

- Awareness Campaign
  - Mission/Goals
  - Public Service Announcements
  - Brochures
  - Photo Exhibit
  - Toolkit: Fact Sheets
- For Patients & Caregivers
  - Definition
  - Symptoms
  - Who's at Risk
  - Possible Causes
  - Diagnosis
  - Brochures
  - Treatment Options
  - How to Talk to Your Doctor
  - Basic Facts/ FAQ
  - Support Groups
- For Healthcare Professionals
  - Definition
  - Symptoms
  - Who's at Risk
  - Diagnosis
  - Brochures
  - Treatment Options / Management Plans
  - Research: New Knowledge & Publications
  - Meetings & Workshops
  - Continuing Education
  - Toolkit: Fact Sheets
  - CDC Research Group
- News & Highlights
- Glossary
- Topic Home

Chronic Fatigue Syndrome > Awareness Campaign >

Photo Exhibit: The Faces of Chronic Fatigue Syndrome

On this page

- Exhibit Description
- Exhibit Venues

Exhibit Description

Adrianne THE SURVIVOR

A CFS photo exhibit called "The Faces of Chronic Fatigue Syndrome" is traveling to cities across the country.

The exhibit features 10 portraits from well-known photographer George Lange. He photographed 8 patients and 2 CFS experts for the traveling exhibit. The huge portrait banners, which are more than 7 feet tall, include quotes from the 10 participants, who describe CFS and the impact it has on patients and their families.

Photos from the exhibit can be seen throughout the CDC Chronic Fatigue Syndrome Web site.

Exhibit Venues

You can see the CFS photo exhibit at the following locations:

Venue	Location	Date
Westfield Meriden	Meriden, CT	June 15-21, 2009
Mayfair Mall	Wauwatosa, WI	May 11-17, 2009
St. Louis Mills	Hazelwood, MO	April 23-26, 2009
Meadowood Mall	Reno, NV	March 9-15, 2009
The Peyton Anderson Health Education Center at The Medical Center of Central Georgia	Macon, GA	September 22-29, 2008
Penn Square	Oklahoma City, OK	September 4-8, 2008
Tacoma Mall	Tacoma, WA	August 14-18, 2008
Great Lakes Mall	Mentor, OH	July 17-21, 2008
The Maryland Science Center	Baltimore, MD	June 23-29, 2008
The San Antonio Public Library	San Antonio, TX	May 16-22, 2008
The Museum of Science & Industry	Tampa, FL	April 21-28, 2008

Page last modified: March 17, 2009
Content source: Division of Viral & Rickettsial Diseases
National Center for Zoonotic, Vector-Borne, and Enteric Diseases (ZVED)

E-mail this page
Printer-friendly version

News & Highlights

- CFS Public Health Research Program Draft 5-year Strategic Plan *NEW*
- CDC Seeks Input to Shape a CFS Strategic Research Plan
- CFS Research Program, External Peer Review (PDF – 46KB)
- New Publication: 1994 CFS Case Definition Criteria Validated
- New Publication Linking Sexual Abuse and CFS
- Current CDC Research
- CME Course
- Chronic Fatigue Registry
- Treatment Guidelines
- Other Federal Resources
- Brochures
- Glossary
- more...

CFS Awareness Campaign

Call to Action - Act Now

- Mission/Goals
- Public Service Announcements
- Brochures
- Photo Exhibit

Paid for by
HHS CDC

CDC Home | Policies and Regulations | Disclaimer | e-Government | FOIA | Contact Us

SAFER·HEALTHIER·PEOPLE™
Centers for Disease Control and Prevention, 1600 Clifton Rd, Atlanta, GA 30333, USA
Tel: 404-639-3311 • CDC Contact Center: 800-CDC-INFO • 888-232-6348 (TTY)

USA.gov Government Made Easy

Department of Health and Human Services

g. Screenshot of web page: *Photo Exhibit*

CDC Home | About CDC | Press Room | A-Z Index | Contact Us

Department of Health and Human Services
Centers for Disease Control and Prevention

CDC en Español

Search: GO

Chronic Fatigue Syndrome

Topic Contents

Awareness Campaign
- Mission/Goals
- Public Service Announcements
- Brochures
- Photo Exhibit
- Toolkit: Fact Sheets

For Patients & Caregivers
- Definition
- Symptoms
- Who's at Risk
- Possible Causes
- Diagnosis
- Brochures
- Treatment Options
- How to Talk to Your Doctor
- Basic Facts/ FAQ
- Support Groups

For Healthcare Professionals
- Definition
- Symptoms
- Who's at Risk
- Diagnosis
- Brochures
- Treatment Options / Management Plans
- Research: New Knowledge & Publications
- Meetings & Workshops
- Continuing Education
- Toolkit: Fact Sheets
- CDC Research Group

News & Highlights

Glossary

Topic Home

//-->

Chronic Fatigue Syndrome > Awareness Campaign >

Public Service Announcements (PSAs)

Public service announcements for the CFS public awareness campaign illustrate the impact of the illness, educate people about how to recognize symptoms and refer people to resources that can help.

On this page:
- Radio
- Television
- Message from Dr. Gerberding

Radio

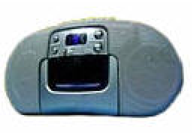

"Get Informed" Radio PSA

- 30 second Radio
- 60 second Radio

Television

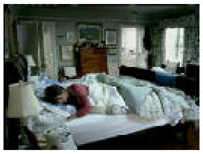

"Missing My Life" Television PSA (30 second)

Message from Dr. Gerberding

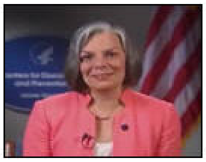

Message from Dr. Julie Gerberding, CDC Director

Page last modified on January 24, 2007

E-mail this page

Printer-friendly version

News & Highlights

- CFS Public Health Research Program Draft 5-year Strategic Plan *NEW*
- CDC Seeks Input to Shape a CFS Strategic Research Plan
- CFS Research Program, External Peer Review (PDF – 46KB)
- New Publication: 1994 CFS Case Definition Criteria Validated
- New Publication Linking Sexual Abuse and CFS
- Current CDC Research
- CME Course
- Chronic Fatigue Registry
- Treatment Guidelines
- Other Federal Resources
- Brochures
- Glossary
- more...

Campaign Photo / Logo

Paid for by

HHS CDC

CDC Home | Policies and Regulations | Disclaimer | e-Government | FOIA | Contact Us

SAFER·HEALTHIER·PEOPLE™
Centers for Disease Control and Prevention, 1600 Clifton Rd, Atlanta, GA 30333, USA
Tel: 404-639-3311 • CDC Contact Center: 800-CDC-INFO • 888-232-6348 (TTY)

USA.gov Government Made Easy

Department of Health and Human Services

h. Screenshot of web page: *PSAs*

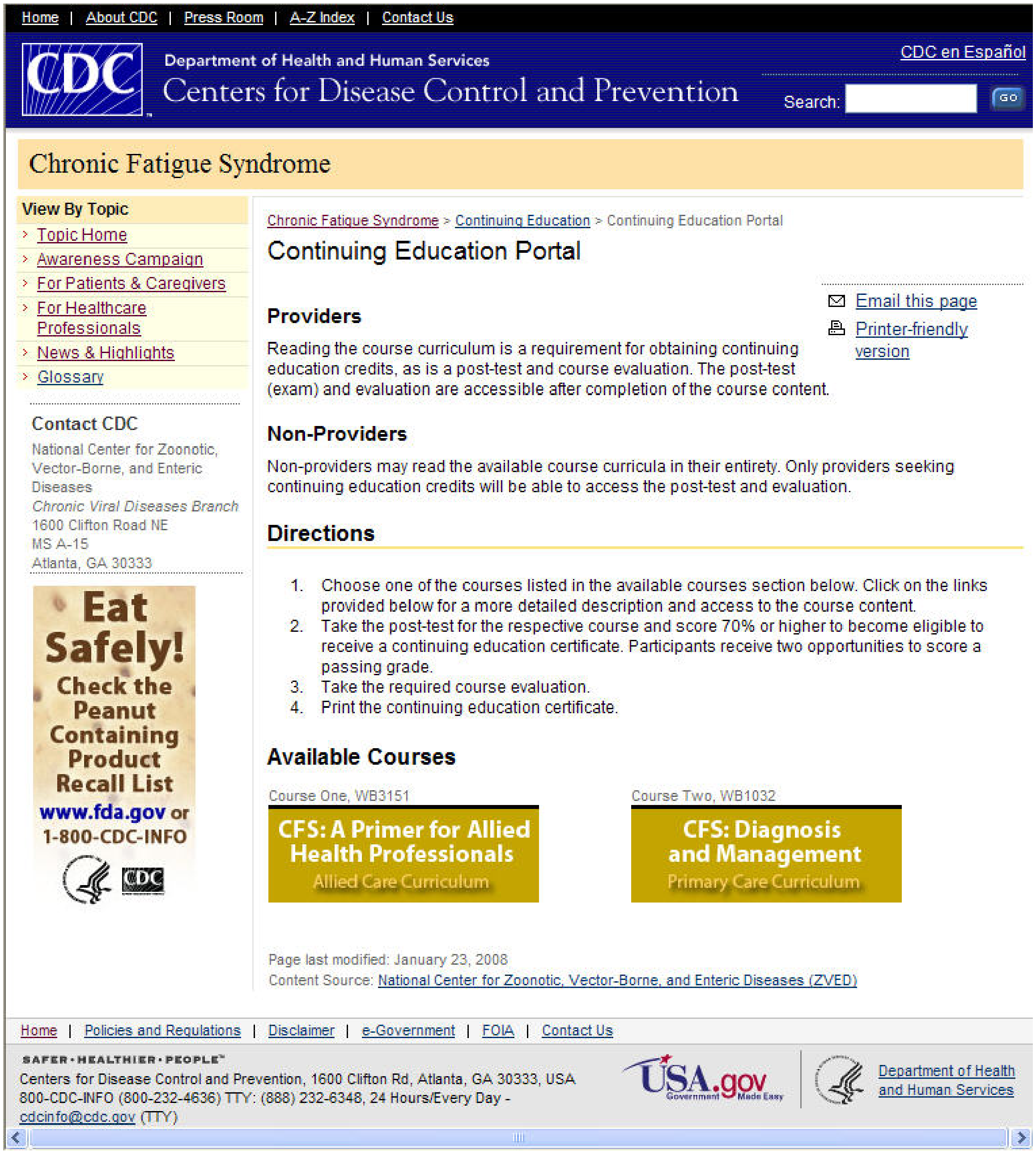

Home | About CDC | Press Room | A-Z Index | Contact Us

CDC

Department of Health and Human Services

Centers for Disease Control and Prevention

CDC en Español

Search: GO

Chronic Fatigue Syndrome

View By Topic

- Topic Home
- Awareness Campaign
- For Patients & Caregivers
- For Healthcare Professionals
- News & Highlights
- Glossary

Contact CDC

National Center for Zoonotic, Vector-Borne, and Enteric Diseases
Chronic Viral Diseases Branch
1600 Clifton Road NE
MS A-15
Atlanta, GA 30333

Eat Safely! Check the Peanut Containing Product Recall List www.fda.gov or 1-800-CDC-INFO

Chronic Fatigue Syndrome > Continuing Education > Continuing Education Portal

Continuing Education Portal

Email this page

Printer-friendly version

Providers

Reading the course curriculum is a requirement for obtaining continuing education credits, as is a post-test and course evaluation. The post-test (exam) and evaluation are accessible after completion of the course content.

Non-Providers

Non-providers may read the available course curricula in their entirety. Only providers seeking continuing education credits will be able to access the post-test and evaluation.

Directions

1. Choose one of the courses listed in the available courses section below. Click on the links provided below for a more detailed description and access to the course content.
2. Take the post-test for the respective course and score 70% or higher to become eligible to receive a continuing education certificate. Participants receive two opportunities to score a passing grade.
3. Take the required course evaluation.
4. Print the continuing education certificate.

Available Courses

Course One, WB3151

CFS: A Primer for Allied Health Professionals
Allied Care Curriculum

Course Two, WB1032

CFS: Diagnosis and Management
Primary Care Curriculum

Page last modified: January 23, 2008
Content Source: National Center for Zoonotic, Vector-Borne, and Enteric Diseases (ZVED)

Home | Policies and Regulations | Disclaimer | e-Government | FOIA | Contact Us

SAFER·HEALTHIER·PEOPLE™
Centers for Disease Control and Prevention, 1600 Clifton Rd, Atlanta, GA 30333, USA
800-CDC-INFO (800-232-4636) TTY: (888) 232-6348, 24 Hours/Every Day - cdcinfo@cdc.gov (TTY)

USA.gov Government Made Easy

Department of Health and Human Services

i. Screenshot of web page: *Continuing Education Portal*

CDC Home | About CDC | Press Room | A-Z Index | Contact Us

CDC en Español

Department of Health and Human Services
Centers for Disease Control and Prevention

Search: GO

Chronic Fatigue Syndrome

Topic Contents

- Awareness Campaign
- For Patients & Caregivers
  - Definition
  - Symptoms
  - Who's at Risk
  - Possible Causes
  - Diagnosis
  - Brochures
  - Treatment Options
  - How to Talk to Your Doctor
  - Basic Facts/ FAQ
  - Support Groups
- For Healthcare Professionals
  - Definition
  - Symptoms
  - Who's at Risk
  - Diagnosis
  - Brochures
  - Treatment Options / Management Plans
  - Research: New Knowledge & Publications
  - Meetings & Workshops
  - Continuing Education
  - Toolkit: Fact Sheets
  - CDC Research Group
- News & Highlights
- Glossary
- Topic Home

Chronic Fatigue Syndrome >

Research: New Knowledge & Publications

On this page:

- Publications
- Studies

Publications

This is a compilation of publications by the CDC CFS Public Health Research Program from 1993 to present. This section includes only publications involving the CDC CFS research group. The National Institutes of Health Trans-NIH Working Group for Research on Chronic Fatigue Syndrome (http://orwh.od.nih.gov/cfs.html*) contains a link to the PubMed database that includes peer reviewed articles on CFS in more than 4,800 biomedical journals over the last decade.

The objective of CDC's CFS research program is to devise control and prevention strategies for CFS The research strategy is based on longitudinal surveillance of defined populations. This avoids the bias of studies based on patients referred from physicians' practices or studies that solicit volunteers. We use random digit dialing to evaluate households for unwellness, detailed telephone interviews of well and unwell members of surveyed household, and then detailed one day clinical evaluations of those identified as possibly having CFS and randomly selected well individuals. The surveyed populations are followed over time (cohort design) to identify incident cases and evaluate the clinical course of the illness. Finally, people identified with CFS and various controls are invited to participate in multi-day in-patient studies. In addition to questionnaire data, we measure clinical parameters (e.g., polysomnography, orthostatic instability), evaluate cognitive function, measure immune status (e.g., cytokines), evaluate neuroendocrine status (e.g., diurnal cortisol profiles), determine allostatic load, evaluate gene expression profiles, measure genetic polymorphisms, and measure the proteome (e.g., SELDI-TOF). Clearly CFS represents a complex illness that involves multiple body systems, includes alterations in homeostatic systems, and results from the combined action of many genes, environmental factors and risk-conferring behavior. The research group is multidisciplinary and includes physicians, epidemiologists, behavioral scientists, statisticians, microbiologists, and mathematicians.

Although somewhat artificial, this compilation of publications from the CDC CFS Public Health Research Group is divided into those dealing primarily with surveillance, defining CFS, studies of causality and risk factors, pathophysiology, clinical aspects, medical management, economic impact and molecular epidemiology. Each article is referenced, includes a summary, and a copy of the abstract as published. Some articles may be directly downloaded and the appropriate URL is noted. Reprints of all the articles are available upon request.

Surveillance Studies

Case Definition

Studies of Causes

Pathophysiology

Clinical Picture

Medical Management

Economic Impact

Molecular Epidemiology Program

Cluster Investigations

Studies

CDC conducted a study of CFS and similar illnesses in 13 counties in Georgia. Interviewers telephoned a randomly selected sample of 17,000 households and asked the selected households a short set of questions to identify household members who may have CFS and similar illnesses.

Results from this study are being prepared for publication.

* Links to non-Federal organizations found at this site are provided solely as a service to our users. These links do not constitute an endorsement of these organizations or their programs by CDC or the Federal Government, and none should be inferred. CDC is not responsible for the content of the individual organization Web pages found at these links.

Page last modified: February 29, 2008
Content source: National Center for Zoonotic, Vector-Borne, and Enteric Diseases (ZVED)

E-mail this page
Printer-friendly version

News & Highlights

- CFS Public Health Research Program Draft 5-year Strategic Plan *NEW*
- CDC Seeks Input to Shape a CFS Strategic Research Plan
- CFS Research Program, External Peer Review (PDF – 46KB)
- New Publication: 1994 CFS Case Definition Criteria Validated
- New Publication Linking Sexual Abuse and CFS
- Current CDC Research
- CME Course
- Chronic Fatigue Registry
- Treatment Guidelines
- Other Federal Resources
- Brochures
- Glossary
- more...

CFS Awareness Campaign

Call to Action - Act Now

- Mission/Goals
- Public Service Announcements
- Brochures
- Photo Exhibit

Paid for by
HHS CDC

CDC Home | Policies and Regulations | Disclaimer | e-Government | FOIA | Contact Us

SAFER·HEALTHIER·PEOPLE™
Centers for Disease Control and Prevention, 1600 Clifton Rd, Atlanta, GA 30333, USA
Tel: 404-639-3311 • CDC Contact Center: 800-CDC-INFO • 888-232-6348 (TTY)

USA.gov Government Made Easy | Department of Health and Human Services

j. Screenshot of web page: *New Knowledge & Publications*

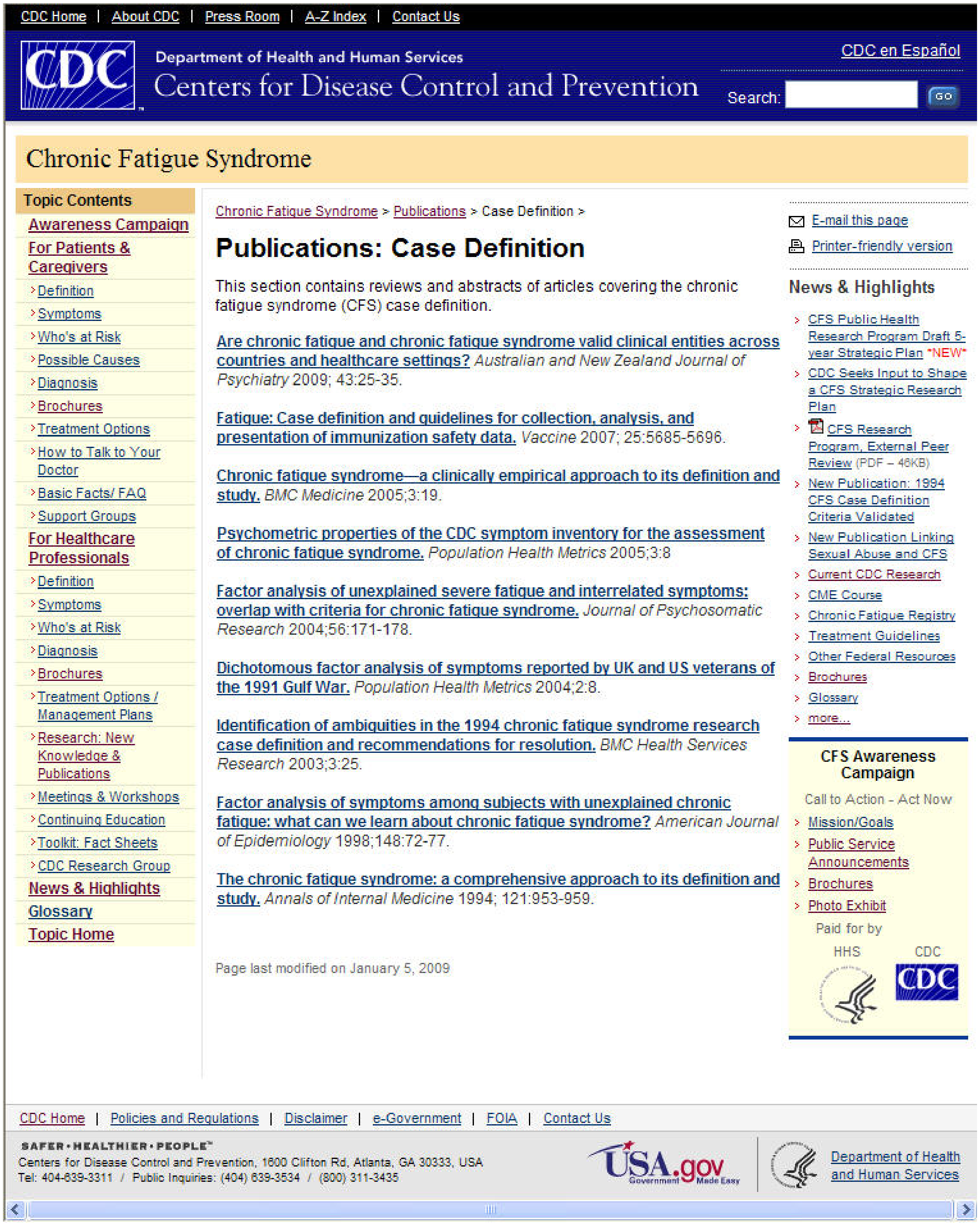

CDC Home | About CDC | Press Room | A-Z Index | Contact Us

CDC Department of Health and Human Services
Centers for Disease Control and Prevention

CDC en Español

Search: GO

Chronic Fatigue Syndrome

Topic Contents

- Awareness Campaign
- For Patients & Caregivers
  - Definition
  - Symptoms
  - Who's at Risk
  - Possible Causes
  - Diagnosis
  - Brochures
  - Treatment Options
  - How to Talk to Your Doctor
  - Basic Facts/ FAQ
  - Support Groups
- For Healthcare Professionals
  - Definition
  - Symptoms
  - Who's at Risk
  - Diagnosis
  - Brochures
  - Treatment Options / Management Plans
  - Research: New Knowledge & Publications
  - Meetings & Workshops
  - Continuing Education
  - Toolkit: Fact Sheets
  - CDC Research Group
- News & Highlights
- Glossary
- Topic Home

Chronic Fatigue Syndrome > Publications > Case Definition >

Publications: Case Definition

This section contains reviews and abstracts of articles covering the chronic fatigue syndrome (CFS) case definition.

Are chronic fatigue and chronic fatigue syndrome valid clinical entities across countries and healthcare settings? *Australian and New Zealand Journal of Psychiatry* 2009; 43:25-35.

Fatigue: Case definition and guidelines for collection, analysis, and presentation of immunization safety data. *Vaccine* 2007; 25:5685-5696.

Chronic fatigue syndrome—a clinically empirical approach to its definition and study. *BMC Medicine* 2005;3:19.

Psychometric properties of the CDC symptom inventory for the assessment of chronic fatigue syndrome. *Population Health Metrics* 2005;3:8

Factor analysis of unexplained severe fatigue and interrelated symptoms: overlap with criteria for chronic fatigue syndrome. *Journal of Psychosomatic Research* 2004;56:171-178.

Dichotomous factor analysis of symptoms reported by UK and US veterans of the 1991 Gulf War. *Population Health Metrics* 2004;2:8.

Identification of ambiguities in the 1994 chronic fatigue syndrome research case definition and recommendations for resolution. *BMC Health Services Research* 2003;3:25.

Factor analysis of symptoms among subjects with unexplained chronic fatigue: what can we learn about chronic fatigue syndrome? *American Journal of Epidemiology* 1998;148:72-77.

The chronic fatigue syndrome: a comprehensive approach to its definition and study. *Annals of Internal Medicine* 1994; 121:953-959.

Page last modified on January 5, 2009

E-mail this page

Printer-friendly version

News & Highlights

- CFS Public Health Research Program Draft 5-year Strategic Plan *NEW*
- CDC Seeks Input to Shape a CFS Strategic Research Plan
- CFS Research Program, External Peer Review (PDF – 46KB)
- New Publication: 1994 CFS Case Definition Criteria Validated
- New Publication Linking Sexual Abuse and CFS
- Current CDC Research
- CME Course
- Chronic Fatigue Registry
- Treatment Guidelines
- Other Federal Resources
- Brochures
- Glossary
- more...

CFS Awareness Campaign

Call to Action - Act Now

- Mission/Goals
- Public Service Announcements
- Brochures
- Photo Exhibit

Paid for by

HHS CDC

CDC Home | Policies and Regulations | Disclaimer | e-Government | FOIA | Contact Us

SAFER·HEALTHIER·PEOPLE™
Centers for Disease Control and Prevention, 1600 Clifton Rd, Atlanta, GA 30333, USA
Tel: 404-639-3311 / Public Inquiries: (404) 639-3534 / (800) 311-3435

USA.gov Government Made Easy

Department of Health and Human Services

k. Screenshot of web page: *Publications: Case Definition*

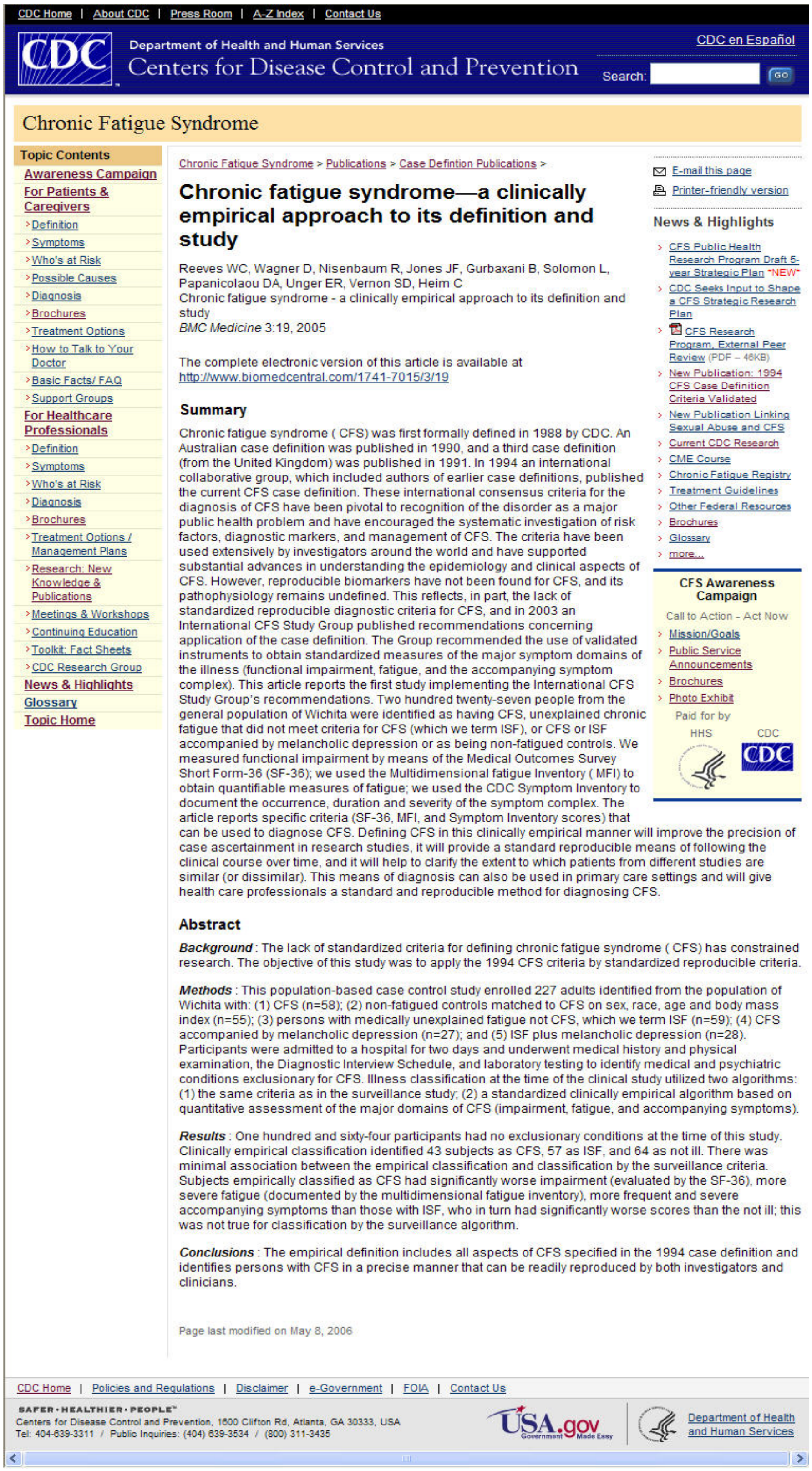

CDC Home | About CDC | Press Room | A-Z Index | Contact Us

Department of Health and Human Services
Centers for Disease Control and Prevention

CDC en Español

Search: GO

Chronic Fatigue Syndrome

Topic Contents

- Awareness Campaign
- For Patients & Caregivers
  - Definition
  - Symptoms
  - Who's at Risk
  - Possible Causes
  - Diagnosis
  - Brochures
  - Treatment Options
  - How to Talk to Your Doctor
  - Basic Facts/ FAQ
  - Support Groups
- For Healthcare Professionals
  - Definition
  - Symptoms
  - Who's at Risk
  - Diagnosis
  - Brochures
  - Treatment Options / Management Plans
  - Research: New Knowledge & Publications
  - Meetings & Workshops
  - Continuing Education
  - Toolkit: Fact Sheets
  - CDC Research Group
- News & Highlights
- Glossary
- Topic Home

Chronic Fatigue Syndrome > Publications > Case Defintion Publications >

Chronic fatigue syndrome—a clinically empirical approach to its definition and study

Reeves WC, Wagner D, Nisenbaum R, Jones JF, Gurbaxani B, Solomon L, Papanicolaou DA, Unger ER, Vernon SD, Heim C
Chronic fatigue syndrome - a clinically empirical approach to its definition and study
BMC Medicine 3:19, 2005

The complete electronic version of this article is available at
http://www.biomedcentral.com/1741-7015/3/19

Summary

Chronic fatigue syndrome (CFS) was first formally defined in 1988 by CDC. An Australian case definition was published in 1990, and a third case definition (from the United Kingdom) was published in 1991. In 1994 an international collaborative group, which included authors of earlier case definitions, published the current CFS case definition. These international consensus criteria for the diagnosis of CFS have been pivotal to recognition of the disorder as a major public health problem and have encouraged the systematic investigation of risk factors, diagnostic markers, and management of CFS. The criteria have been used extensively by investigators around the world and have supported substantial advances in understanding the epidemiology and clinical aspects of CFS. However, reproducible biomarkers have not been found for CFS, and its pathophysiology remains undefined. This reflects, in part, the lack of standardized reproducible diagnostic criteria for CFS, and in 2003 an International CFS Study Group published recommendations concerning application of the case definition. The Group recommended the use of validated instruments to obtain standardized measures of the major symptom domains of the illness (functional impairment, fatigue, and the accompanying symptom complex). This article reports the first study implementing the International CFS Study Group's recommendations. Two hundred twenty-seven people from the general population of Wichita were identified as having CFS, unexplained chronic fatigue that did not meet criteria for CFS (which we term ISF), or CFS or ISF accompanied by melancholic depression or as being non-fatigued controls. We measured functional impairment by means of the Medical Outcomes Survey Short Form-36 (SF-36); we used the Multidimensional fatigue Inventory (MFI) to obtain quantifiable measures of fatigue; we used the CDC Symptom Inventory to document the occurrence, duration and severity of the symptom complex. The article reports specific criteria (SF-36, MFI, and Symptom Inventory scores) that can be used to diagnose CFS. Defining CFS in this clinically empirical manner will improve the precision of case ascertainment in research studies, it will provide a standard reproducible means of following the clinical course over time, and it will help to clarify the extent to which patients from different studies are similar (or dissimilar). This means of diagnosis can also be used in primary care settings and will give health care professionals a standard and reproducible method for diagnosing CFS.

Abstract

Background : The lack of standardized criteria for defining chronic fatigue syndrome (CFS) has constrained research. The objective of this study was to apply the 1994 CFS criteria by standardized reproducible criteria.

Methods : This population-based case control study enrolled 227 adults identified from the population of Wichita with: (1) CFS (n=58); (2) non-fatigued controls matched to CFS on sex, race, age and body mass index (n=55); (3) persons with medically unexplained fatigue not CFS, which we term ISF (n=59); (4) CFS accompanied by melancholic depression (n=27); and (5) ISF plus melancholic depression (n=28). Participants were admitted to a hospital for two days and underwent medical history and physical examination, the Diagnostic Interview Schedule, and laboratory testing to identify medical and psychiatric conditions exclusionary for CFS. Illness classification at the time of the clinical study utilized two algorithms: (1) the same criteria as in the surveillance study; (2) a standardized clinically empirical algorithm based on quantitative assessment of the major domains of CFS (impairment, fatigue, and accompanying symptoms).

Results : One hundred and sixty-four participants had no exclusionary conditions at the time of this study. Clinically empirical classification identified 43 subjects as CFS, 57 as ISF, and 64 as not ill. There was minimal association between the empirical classification and classification by the surveillance criteria. Subjects empirically classified as CFS had significantly worse impairment (evaluated by the SF-36), more severe fatigue (documented by the multidimensional fatigue inventory), more frequent and severe accompanying symptoms than those with ISF, who in turn had significantly worse scores than the not ill; this was not true for classification by the surveillance algorithm.

Conclusions : The empirical definition includes all aspects of CFS specified in the 1994 case definition and identifies persons with CFS in a precise manner that can be readily reproduced by both investigators and clinicians.

Page last modified on May 8, 2006

E-mail this page
Printer-friendly version

News & Highlights

- CFS Public Health Research Program Draft 5-year Strategic Plan *NEW*
- CDC Seeks Input to Shape a CFS Strategic Research Plan
- CFS Research Program, External Peer Review (PDF – 46KB)
- New Publication: 1994 CFS Case Definition Criteria Validated
- New Publication Linking Sexual Abuse and CFS
- Current CDC Research
- CME Course
- Chronic Fatigue Registry
- Treatment Guidelines
- Other Federal Resources
- Brochures
- Glossary
- more...

CFS Awareness Campaign

Call to Action - Act Now

- Mission/Goals
- Public Service Announcements
- Brochures
- Photo Exhibit

Paid for by
HHS CDC

CDC Home | Policies and Regulations | Disclaimer | e-Government | FOIA | Contact Us

SAFER · HEALTHIER · PEOPLE™
Centers for Disease Control and Prevention, 1600 Clifton Rd, Atlanta, GA 30333, USA
Tel: 404-639-3311 / Public Inquiries: (404) 639-3534 / (800) 311-3435

USA.gov Government Made Easy

Department of Health and Human Services

l. Screenshot of web page for an individual publication
